# Endocrine disruptors affect the expression of estrogen receptor genes and proteins in the white cloud mountain minnow *Tanichthys albonubes* (Teleostei: Cyprinidae)

**DOI:** 10.3389/fphys.2022.1020840

**Published:** 2022-10-12

**Authors:** Chen Ke, Chen Meilin, Ma Guangzhi, Fan Yuqin, Liu Lin, Chen Weiting

**Affiliations:** ^1^ Guangdong Provincial Key Laboratory of Conservation and Precision Utilization of Characteristic Agricultural Resources in Mountainous Areas, School of Life Sciences, Jiaying University, Meizhou, China; ^2^ School of Life Sciences, South China Normal University, Guangzhou, China

**Keywords:** estrogen receptor, endocrine disrupting chemicals, Tanichthys albonubes, testis, brain

## Abstract

The endocrine disruptor chemicals (EDCs) are ubiquitous in the environment, and it has raised wide public concern because of the dangers of EDCs for living organisms and the environment. In order to comparatively study the effects of EDCs [17-α-ethinylestradiol (EE_2_), Bisphenol A (BPA) and Nonylphenol (NP)] on the expression of estrogen receptors (ERs: *erα, erβ1, and erβ2*) at mRNA and protein level, total 520 adult *Tanichthys albonubes* were exposed to E_2_, EE_2_, BPA and NP with three concentrations respectively: EE_2_ (1, 5, 25 ng/l), NP (10, 50, 250 μg/l), BPA (100, 500, 2,500 μg/l) for 28 days, E_2_ (2, 20, 200 ng/l) being as the positive control. After treatment, the brain, eye, gill, heart, liver, gut, kidney, muscle, testis, and ovary were collected, following by the real-time quantitative PCR (RT-qPCR) and western blot methods to detect the expression levels of *erα*, *erβ1*, and *erβ2* in *T.albonubes* at mRNA and protein level. Our results showed that high expression of *terα (t means T.albonubes), terβ1*, and *terβ2* were detected in liver, while *terβ1* and *terβ2* mainly expressed in the liver, intestine, kidney, muscle and testis. EE_2_, BPA, and NP treatment all up-regulated the expression of *terα, terβ1*, and *terβ2* in the brain, liver, and testis, but with some variations. Similar to mRNA level, both TERα and TERβ were up-regulated by all the EE_2_, BPA, and NP treatment with dose-dependent effect. In conclusion, the responses of ERs of *T.albonubes* to the EDCs present measurability and susceptibility, which make it possible for *T. albonubes* to be an efficient biomarker to monitor and evaluate the pollution of endocrine disrupting chemicals in water environment.

## 1 Introduction

The rapid industrialization of our planet over previous decades has resulted in the release of various newly synthesized chemical substances into the environment, and some of the most of them concerning are environmental estrogens (EEs), which are one of the main types of environmental endocrine disruptor chemicals (EDCs). EDCs are ubiquitous in the environment and vary substantially in chemical structure, but most end up in water bodies via both atmospheric circulation and water circulation. EDCs can have negative effects on aquatic animals due to their high lipid solubility, resistance to degradation, high toxicity, and ease with which they can become enriched. The interactions of EDCs with other compounds can also amplify their deleterious effects (Silva et al., 2019). It has raised wide public concern because of the dangers of EDCs for living organisms and the environment (Colborn et al., 1993; Song et al., 2022). For example, EEs treatment can result in feminization, hermaphroditism, and sex reversion, which can reduce the reproductive capacity of populations and increase extinction risk (Arcand-Hoy et al., 1998; Sanderson et al., 2004). A previous study has indicated that a sex reversal in medaka might be caused by exposure to high concentrations of 17-α-ethinylestradiol (EE2) (Scholz and Gutzeit 2000). EE2 exposure has been shown to negatively affect the development and reproductive health of *Gobiocypris rarus* (Zha et al., 2008), as well as the gonadal differentiation and clutch size of zebrafish (Brion et al., 2004). Nonylphenol (NP) and bisphenol A (BPA) can also have deleterious effects on the endocrine system of fish; for example, these substances can reduce the size of the testis, stimulate the apoptosis of spermatocytes, or lead to the appearance of oocytes in males (Kashiwada et al., 2002; Weber et al., 2002).

The above mentioned estrogenic effects of EDCs were mediated by the estrogen receptors (ERs), including *erα, erβ1*, and *erβ2*. However, the ERs showed different responses to those EDCs. Exposure of male medaka to NP and BPA increased the expression level of *erα* in liver (Yamaguchi et al., 2005). It is further confirmed that the feminizing effect of BPA on sexual differentiation was dependent on ERs by using ERs-null mutant zebrafish (Song et al., 2020). In *Tilapia*, NP could up-regulate the expression of both *erα* and *erβ* (Celino-Brady et al., 2019), while it decreased the expression of both *erα* and *erβ* in *Rivulus marmoratus* (Seo et al., 2006). In Salmon, short-term exposure of EE2, but not NP, could up-regulate the *erα* expression, while long-term treatment with EE2 and NP decreased the expression level of *erα* (Breves et al., 2018).

Therefore, further study is necessary to systematically reveal the mechanisms of how the ERs respond to the EDCs. *Tanichtys albonubes*, a freshwater fish native to China that is highly sensitive to environmental changes and has become a model organism for aquatic ecotoxicological studies (Wang and Fang, 2006; Wang et al., 2010; Liu et al., 2012). Here, we used *T. albonubes* as a model to comparatively study the responses of *erα, erβ1, and erβ2* to the EE2, NP and BPA treatment at mRNA and protein levels by using ecotoxicogenomic approaches, and used E_2_ as the positive control. Our results will provide new insights into the mechanism underlying the biological effects of EDCs, and have important implications for both environmental evaluation and monitoring.

## 2 Materials and methods

### 2.1 Chemicals

Chemicals purchased from Sigma-Aldrich Chem (Shanghai, China) include E2 (98% purity), EE_2_ (98% purity), BPA (97% purity), and NP (99% purity). All other chemicals (all analytical grade) were obtained from commercial sources.

### 2.2 Animals and captive care

*T. albonubes* were raised in fish tank in the Laboratory of Animal Resources Protection and Utilization, School of Life Sciences, South China Normal University. Healthy individuals (20 females, weight 0.27 ± 0.07 g, body length 2.59 ± 0.16 cm; 540 males, weight 0.31 ± 0 g, body length 2.72 ± 0.14 cm) were used in experiments. The fish were maintained under a 14:10 light: dark photoperiod and a temperature of 25 ± 1°C; they were fed a commercial diet twice a day.

### 2.3 Patterns of ER gene expression among tissues

After *T. albonubes* reached 7 months of age, fish were euthanized by terminal anesthesia with ice; ovary, testis, liver, brain, intestines, gill, heart, eye, and muscle were extracted from 15 female and male fish and placed into 1.5 ml centrifuge tubes. These tissues were treated with diethyl pyrocarbonate and immediately placed in liquid nitrogen. The samples were then transferred to a freezer (–80°C) until subsequent use.

### 2.4 Exposure to EDCs

After acclimation for 14 days, 7-month-old male *T. albonubes* were exposed to E_2_ (2, 20, and 200 ng/l), EE_2_ (1, 5, and 25 ng/l), NP (10, 50, and 250 μg/l), and BPA (100, 500, and 2,500 μg/l) dissolved in dimethyl sulfoxide (DMSO) in 10 L glass tanks for 28 days; individuals exposed to pure DMSO solvent for the same period were used as the negative control. The used concentrations of those drugs were according to the previous reports (Wang et al., 2011; Zhang et al., 2012) and our preliminary test. The exposure test consisted of 13 groups including the control, and there were two replicates for each group. A total of 26 identical fish tanks were added to 6 L of aerated tap water and 20 7-month-old male fish were added per tank. The fish were fed water fleas twice a day and maintained under a photoperiod of 14 h light: 10 h dark in a climate-controlled room at 25 ± 0.5°C. During the exposure period, half of the water was renewed daily with new EDCs.

### 2.5 RNA extraction and reverse transcription

Total RNA was isolated using Trizol Reagent (Invitrogen) and treated with RNase-free DNase. RNA quality was evaluated using a NanoDrop One pectrophotometer with an A260 nm/A280 nm ratio from 1.8 to 2.0 and the integrity of the 28 and 18 S RNA bands on 1% agarose gels. The cDNA templates were synthesized from 3 µg of DNase-treated total RNA using oligo primers and M-MLV reverse transcriptase (Invitrogen) in a final volume of 20 μl.

### 2.6 Real-time qPCR analysis

Real-time quantitative polymerase chain reaction (RT-qPCR) with the specific primers was used to determine the expression levels of *β-actin* (F-GGAACCGCTGCCTCTTCT TC, R-GCCGCAAGATTCC ATACCAA), *terα* (F-TGCGTAT GAACAGATAGTACCCTTA, R-CGAGAGTTTGTGGGCAGT GG), *terβ1(*F-AACGGTCAAGAAATCACAAATGG, R-GAGT TTGGGGTGGGGCTTT), and *terβ2* (F-CCACCTCATAGCC AAGTTTCATC, R-GTGGTGTAAGTTCCGTCCAAGTC) in the brain, liver, intestine, muscle, gill, kidney, heart, eye, testis, and ovary tissue of *T. albonubes*. The PCR reaction protocol was 40 cycles of 95°C for 30s, 60°C for 30s, and 72°C for 35 s. Signal detection was set at 72°C.

### 2.7 Western blot analysis

Total protein was extracted from frozen testis tissue using sodium dodecyl sulfate (SDS) sample buffer. After separating the eluted proteins using SDS polyacrylamide gel electrophoresis, western blotting was conducted using the anti-ERα (cat. sc-8002) and anti-ERβ antibodies (cat. sc-53494) (Santa Cruz Biotechnology, Texas, United States) at 1:1000 dilution. Enhanced chemiluminescence (Transgene, China) was used to identify the antibody-reactive bands, and the antibody-reactive bands were detected using ChemiScope5300 (Clinx, Shanghai, China). Chemi analysis was used to quantify the intensity of the bands (Clinx, Shanghai, China).

### 2.8 Statistical analysis

All data were expressed as mean ± standard error. The significance of differences between groups were determined using one-way analysis of variance in SPSS 16.0 software; the threshold for statistical significance was *p* < 0.05.

## 3 Results

### 3.1 Expression of *terα, terβ1*, and *terβ2* in *T. albonubes*

**FIGURE 1:**
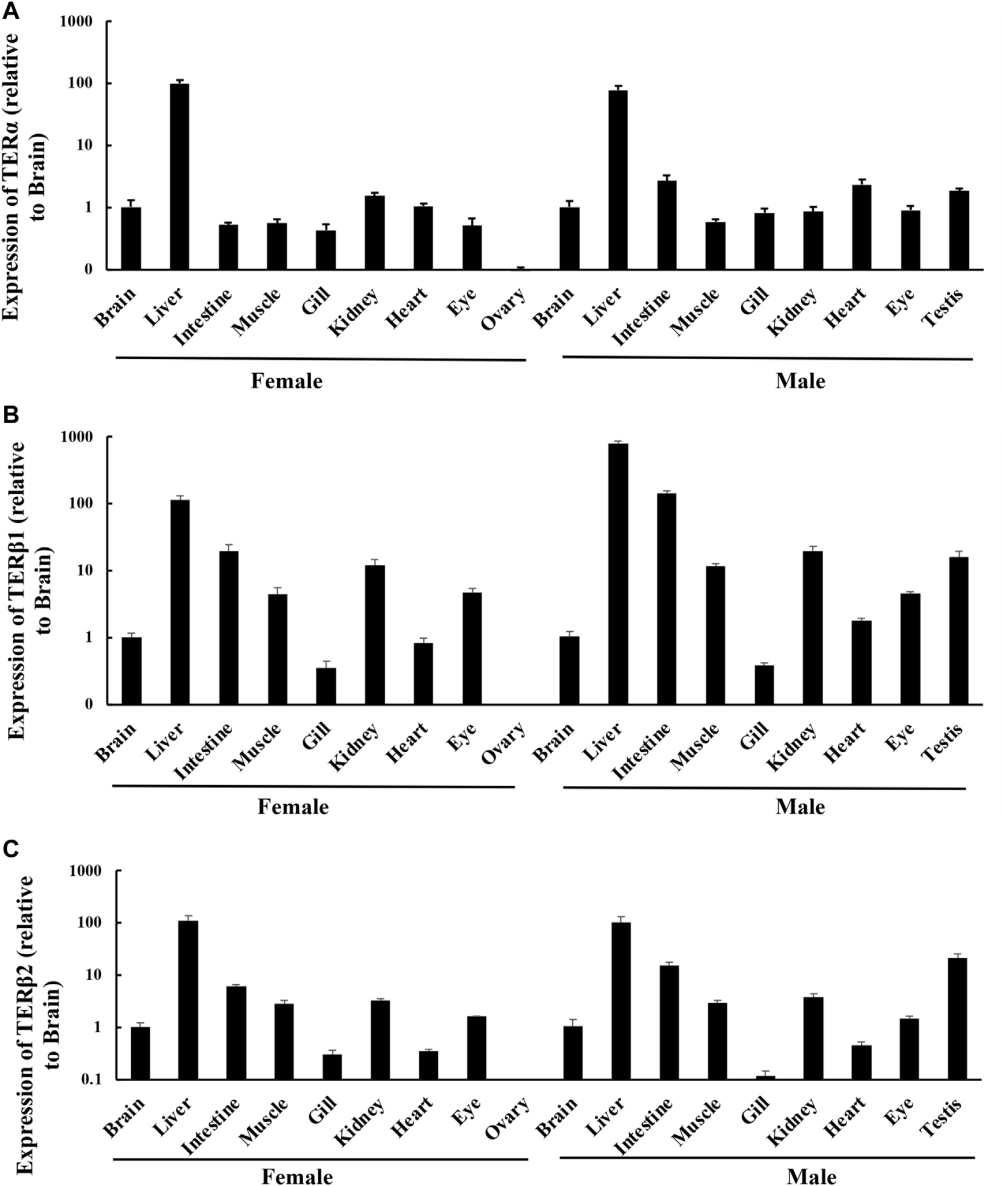
Expression levels of ERs in different tissues in adult *T. albonubes*. Expression of *terα* **(A)**, *terβ1* **(B)**, and *terβ2* **(C)**. The expression of ERs in the different tissues is shown relative to the brain of females and males. The data are expressed as mean ± standard error of 15 female fish and 15 male fish.

RT-qPCR was used to determine the expression levels of *terα*, *terβ1*, and *terβ2* in the brain, liver, intestine, muscle, gill, kidney, heart, eye, testis, and ovary tissue of *T. albonubes*. *Terα* was highly expressed in the liver tissue of male and female fish, and its expression level was much lower in the other tissues (Figure 1A). *Terβ1* and *terβ2* were expressed in all tissues, but they were highly expressed in liver, intestine, muscle, kidney, and testis tissues and weakly expressed in ovary tissue in males and females (Figure 1B,C).

### 3.2 EE_2_, BPA, and NP up-regulated the expression of *terα, terβ1*, and *terβ2* in the brain, liver, and testis

#### 3.2.1 Effects of EDCs on the expression of *terα, terβ1*, and *terβ2* in the brain of *T. albonubes*

**FIGURE 2:**
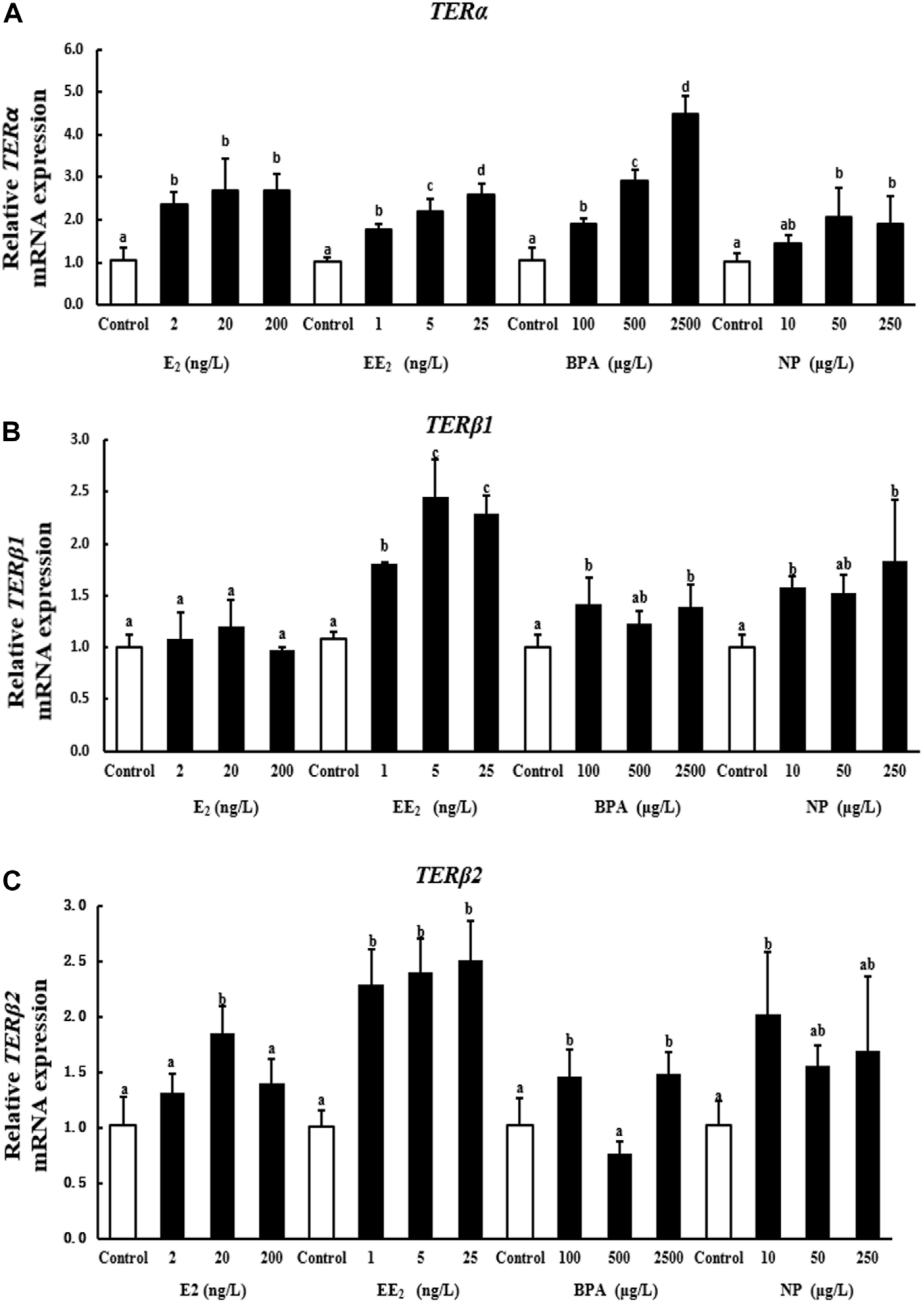
Effects of EDCs on the expression levels of *terα* **(A)**, *terβ1* **(B)**, and *terβ2* **(C)** in the brain of *T. albonubes*. Columns with different letters are significantly different at *p* < 0.05 according to one-way ANOVA followed by Dunnett’s test.

A 28-days exposure experiment revealed that the expression of *terα* in the brain of *T. albonubes* increased with the level of EDC exposure. Significant differences were observed between all exposure groups and the control, with the exception of the 10 μg/l NP exposure group (Figure 2A).

There was no significant difference in the expression of *terβ1* in the E_2_ exposure group. However, the expression of *terβ1* was significantly higher in the EE_2_ exposure group than control. The expression of *terβ1* in brain was also increased with the high dosage of BPA and NP exposure (Figure 2B).

All of the tested EDCs, as well as the E2, increased the expression of *terβ2* in the brain of *T. albonubes*, except the NP treatment. The expression of *terβ2* was significantly higher in the low NP exposure group while no significant differences in higher dosage treatment (Figure 2C).

#### 3.2.2 Effects of EDCs on the expression of *terα, terβ1*, and *terβ2* in the liver of *T. albonubes*

**FIGURE 3:**
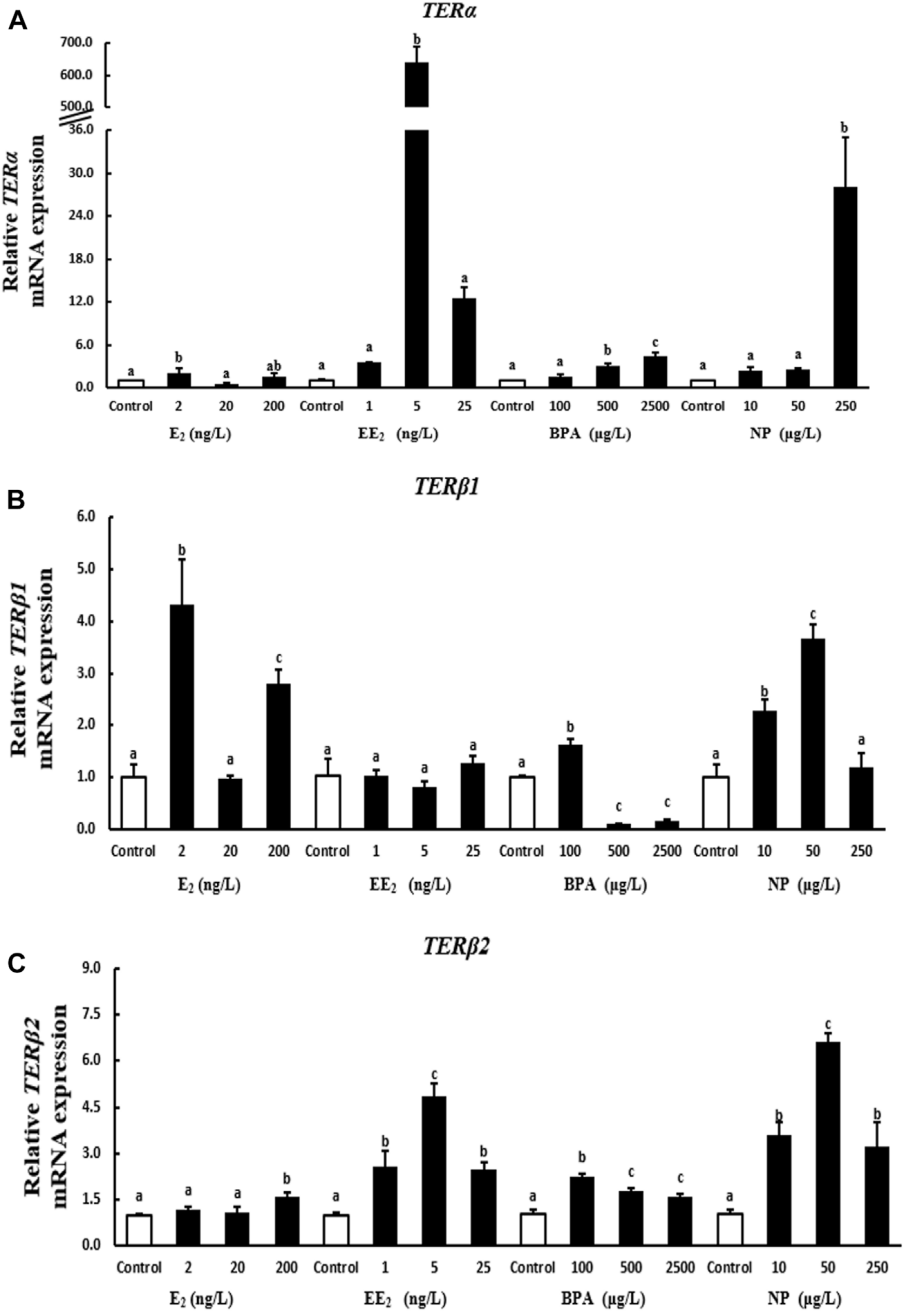
Effects of EDCs on the expression of *terα* **(A)**, *terβ1* **(B)**, and *terβ2* **(C)** in the liver of *T. albonu*bes. Columns with different letters are significantly different at *p* < 0.05 according to one-way ANOVA followed by Dunnett’s test.

A 28-days exposure experiment revealed that the expression of *terα* in liver was significantly higher in the 2 ng/l E_2_ exposure group compared with the control group. By contrast, the expression of *terα* was reduced in the 20 ng/l E_2_ exposure group, and exposure to 200 ng/l E_2_ had no significant effect on *terα* expression. The expression of *terα* was significantly increased by EE_2_ exposure, indicating that *terα* was more sensitive to EE_2_. *Terα* expression was significantly higher in the high BPA and NP exposure groups than in the control group (Figure 3A).

The expression of the *terβ1* gene in liver was significantly higher in the 2 ng/l and 200 ng/l E_2_ exposure groups compared with the control group. There were no significant effects of each EE_2_ exposure group on the expression of *terβ1* in the liver. The expression of *terβ1* was significantly increased in the 100 μg/l BPA exposure group, while it were dramatically decreased at high concentration treatment groups. The expression of *terβ1* was significantly higher in the 10 μg/l and 50 μg/l NP compared with the control group (Figure 3B).

The expression of *terβ2* was significantly higher in the 200 ng/l E_2_ exposure group than in the control group in liver, and no significant differences in *terβ2* expression in liver were observed between the low E_2_ exposure groups and the control group. However, the expression of *terβ2* in liver was significantly higher in the EE_2_, BPA, and NP exposure groups when compared to the control group (Figure 3C).

#### 3.2.3 Effects of EDCs on the expression of *terα, terβ1*, and *terβ2* in the testis of *T. albonubes*

**FIGURE 4:**
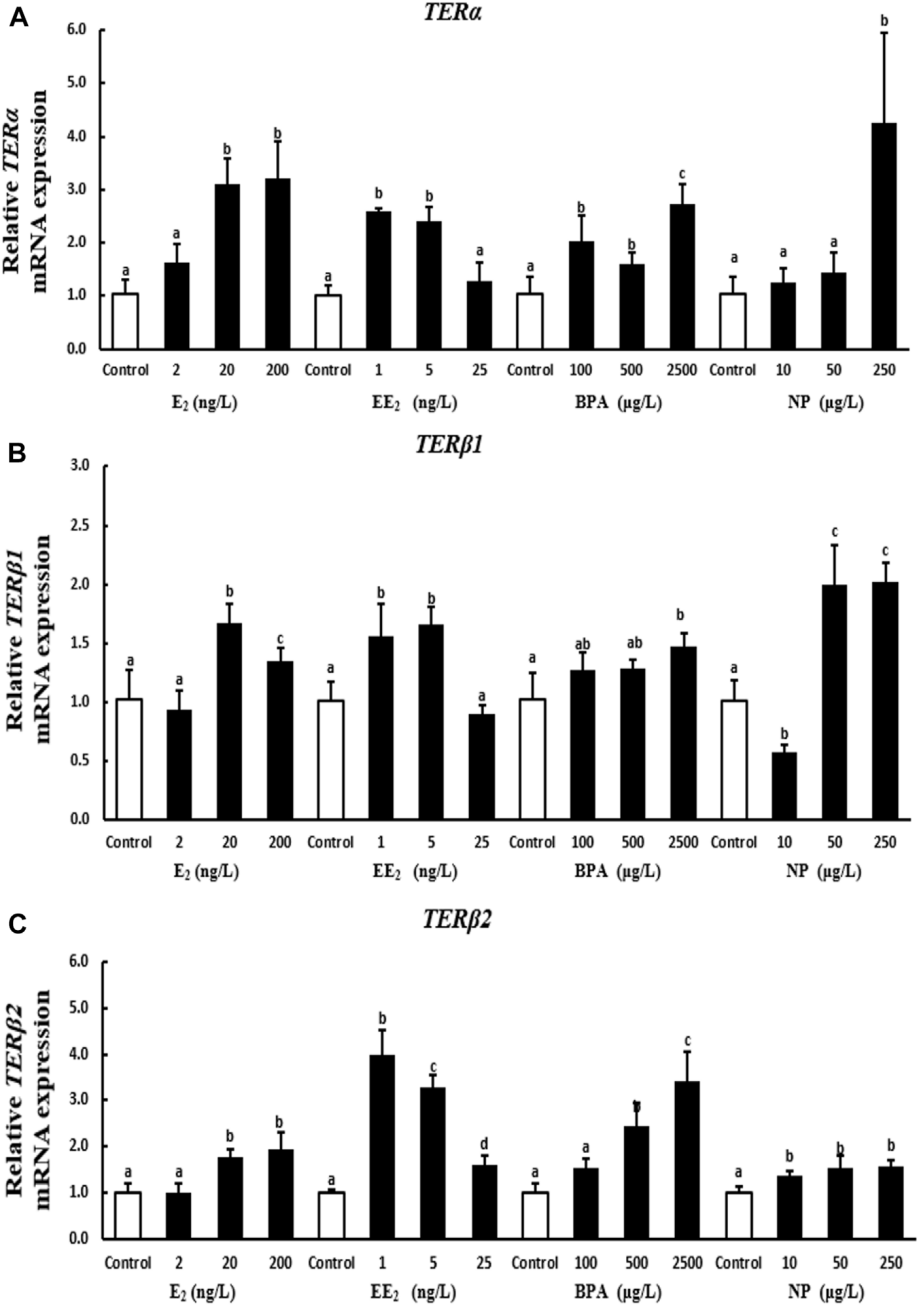
Effects of EDCs on the expression of *terα* **(A)**, *terβ1* **(B)**, and *terβ2* **(C)** in the testis of *T. albonu*bes. Columns with different letters represent the significant difference at *p* < 0.05 according to one-way ANOVA followed by Dunnett’s test.

A 28-days exposure experiment revealed that the expression of *terα* in testis increased with the level of E_2_ and NP exposure. *Terα* expression was significantly higher in the high E_2_ and NP exposure groups than in the control group. The expression of *terα* decreased with the level of EE_2_ exposure. The expression of *terα* in testis was higher under EE_2_ exposure compared with the control group, with the exception of the highest concentration. The expression of *terα* in testis tissue was significantly higher in the BPA exposure groups compared with the control (Figure 4A).

The expression of *terβ1* in testis was significantly higher in the 20 ng/l and 200 ng/l E_2_ exposure groups compared with the control group. The expression of *terβ1* in testis was significantly higher in the low and medium EE_2_ exposure groups compared with the control group, and no significant differences were observed in the expression of *terβ1* between the high EE_2_ exposure group and the control group. The expression of *terβ1* in testis increased after BPA exposure. There was a highly significant difference in the expression of *terβ1* between the high BPA exposure group and the control group. The expression of *terβ1* first decreased while increased when increased the concentration of NP in testis (Figure 4B).

The expression of *terβ2* in testis increased with the level of E_2_, BPA, and NP exposure; however, the expression of *terβ2* was not significantly higher in the 2 ng/l EE_2_ and 100 μg/l BPA exposure groups in testis compared with the control group. The expression of *terβ2* was significantly higher in all other exposure groups compared with the control. The expression of *terβ2* in testis was significantly higher in the EE_2_ exposure groups than in the control group, and *terβ2* expression first increased and then decreased with the level of EE_2_ exposure (Figure 4C).

### 3.3 EE_2_, BPA, and NP up-regulated the expression of the proteins encoded by *terα*, *terβ1*, and *terβ2* in testis

**FIGURE 5:**
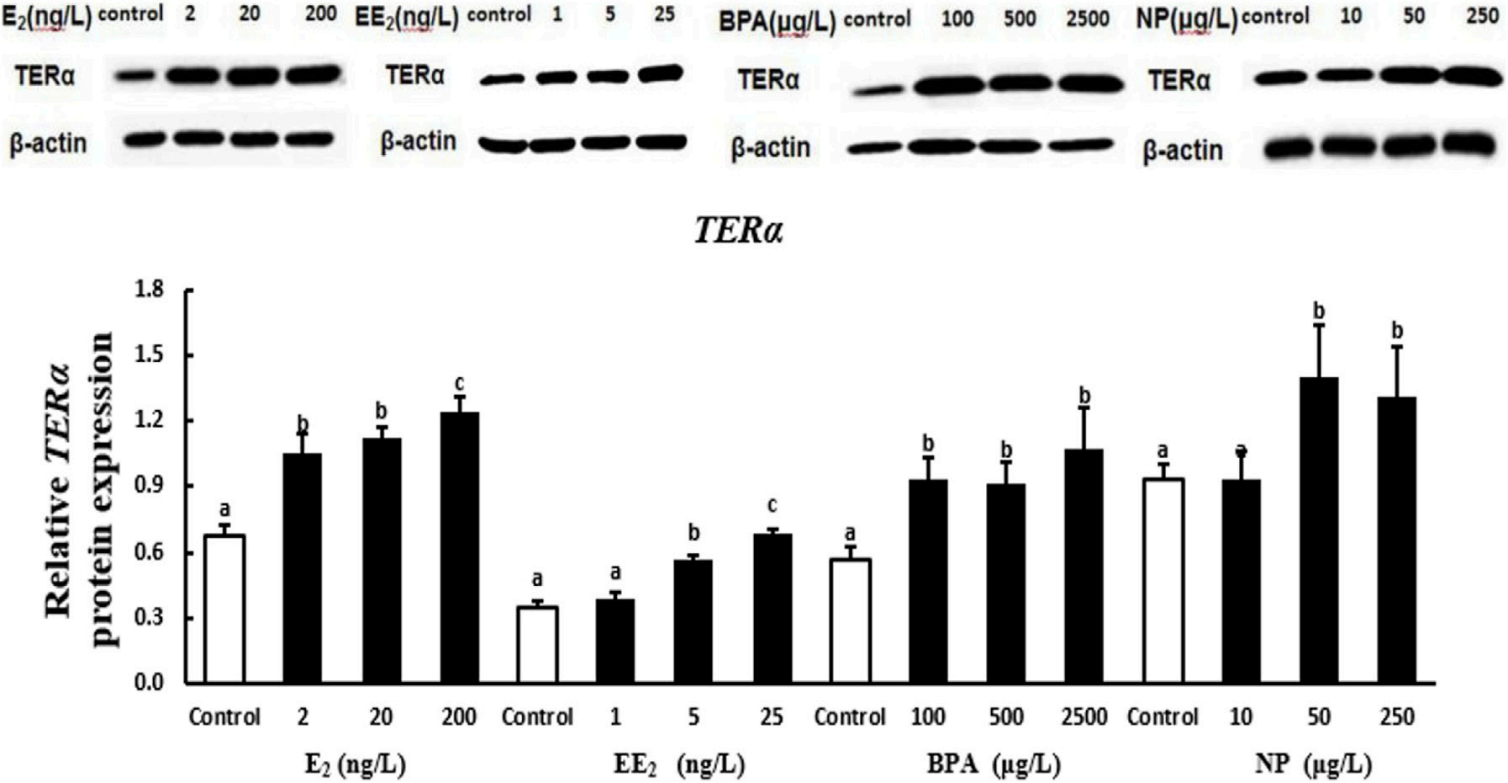
Effects of EDCs on the level of the protein encoded by *terα* in the testis of *T. albonu*bes. Columns with different letters are significantly different at *p* < 0.05 according to one-way ANOVA followed by Dunnett’s test.

At the protein level, the expression of TERα in the testis of *T. albonubes* increased with the level of E_2_ and EE_2_ exposure. Significantly higher expression of TERα was observed in all E_2_ and the medium and high concentrate EE_2_ exposure groups compared with the control. The expression of TERα was higher in the BPA exposure groups compared with the control, and the expression of TERα was highest in the high BPA exposure group. The expression of TERα was significantly higher in the medium and high NP exposure groups compared with the control, and there was no significant difference between the low NP exposure and the control group (Figure 5).

**FIGURE 6:**
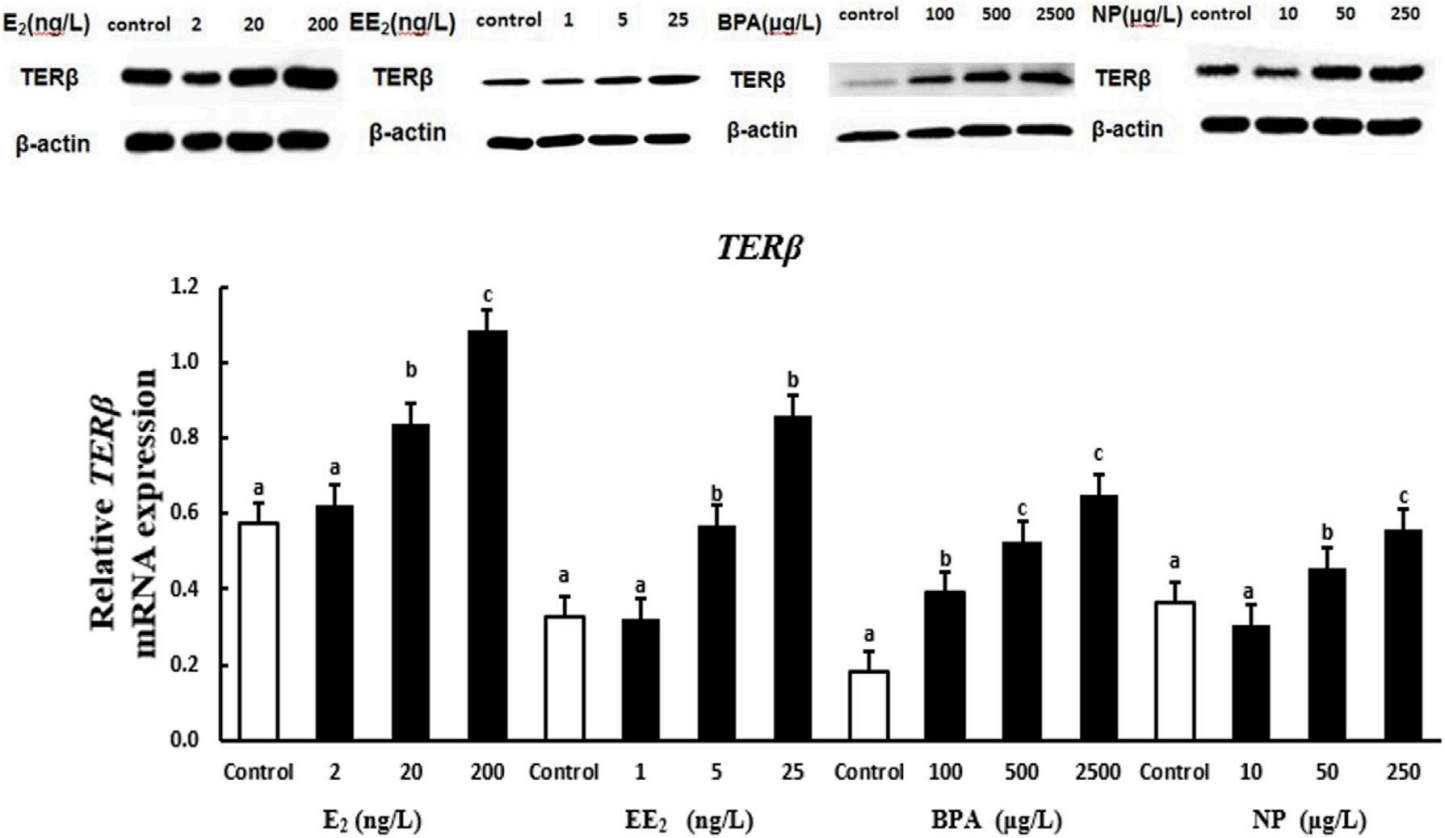
Effects of EDCs on the level of the protein encoded by *terβ* in the testis tissue of *T. albonu*bes. Columns with different letters are significantly different at *p* < 0.05 according to one-way ANOVA followed by Dunnett’s test.

The expression of the protein encoded by *terβ* in the testis of *T. albonubes* increased with the level of E_2_, EE_2_, and BPA exposure. There were significant differences in the expression of the protein encoded by *terβ* between all BPA exposure groups and the control group, and significant differences in expression between the control and the E_2_ and EE_2_ exposure groups were only observed for exposure at medium and high concentrations. The expression of the protein encoded by *terβ* in the testis tissue of *T. albonubes* first increased and then decreased with the level of NP exposure. The expression of the protein encoded by *terβ* was significantly higher in the medium and high NP exposure groups compared with the control group, and no significant differences were observed between the control group and the low NP exposure group (Figure 6).

## 4 Discussion

RT-qPCR was used to quantify the expression of ER genes in several tissues of *T. albonubes*. ER genes were expressed in all tissues of male and female fish, indicating that these ER genes play a role in various processes; this finding is consistent with the results of several previous studies of bony fish (Menuet et al., 2002; Wang et al., 2005; Pinto et al., 2006; Wang et al., 2011). The expression levels of the three ER genes were higher in the liver of female fish compared with other tissues, which is consistent with the results of a previous study of rainbow trout (Nagler et al., 2007), and this pattern might be related to vitellogenin (vtg) synthesis, because Meucci and Arukwe., 2006 found that the expression of *ERα* and *vtg* in the liver is correlated in *Salmo salar* (Meucci and Arukwe 2006). This suggests that ERs mediate important functions of estrogens and facilitate physiological reactions in organisms.

Several previous studies have indicated that the expression levels of ER genes in the male testis can vary(Sharpe 1998; O'Donnell et al., 2001), which challenges the traditional idea that estrogen is a female-specific hormone. In Wang et al., 2011 (Wang et al., 2011)and our study, the high expression of three ER genes in the testis tissue of *T. albonubes* suggests that ERs play important roles in testis function and sperm generation. All three ER genes were highly expressed in the testis tissue and weakly expressed in the ovary tissue, which is in contrast to patterns observed in the expression of ERs in other bony fish species (Zhang et al., 2012). Additional studies are needed to clarify the reasons underlying this inconsistent pattern.

E_2_ is an endogenous estrogen, and Menuet et al. (2004) suggested that the increased expression of *ERα* in zebrafish is induced by self-regulation under E_2_ stimulation (Menuet et al., 2004). An analysis of the upstream promoter sequence of the *ERα* gene showed that it contains a 1/2ERE and an incomplete ERE element (including three mutation sites), whereas the ER genes in *T. albonubes* contain an ERE element. Under E_2_ exposure, the increased expression of ER genes in *T. albonubes* might be related to self-regulation; however, additional experiments are needed to verify this possibility.

EE_2_ is an exogenous hormone with strong estrogenic effects, and it can affect the expression of genes involved in sex differentiation, sex determination, and reproduction. We found that all three concentrations of EE_2_ (1 ng/L, 5 ng/l, and 25 ng/l) can increase the expression of *TERα* and *TERβ2* in liver, testis, and brain tissue. Patterns of variation in the expression of *TERβ2* and *TERα* were similar under exposure to different concentrations of EE_2_. The expression of ER genes in testis tissue was increased by exposure to 1 ng/lEE2, and the most significant increase in the expression of ER genes in liver tissue was induced by exposure to 5 ng/l EE_2_. The expression of ER genes in the brain increased as the concentration of EE_2_ increased. This indicates that testis tissue is highly sensitive to EE_2_ exposure, followed by liver and brain tissue. The high responsiveness of ER genes to EE_2_ in liver and testis tissue suggests that these genes could be used as sensitive biomarkers for the detection of EE_2_ in aquatic environments.

Although BPA has a weak estrogenic effect, it significantly affected the expression of *TERα*, *TERβ1*, and *TERβ2* in *T. albonubes*. Previous studies have revealed an affinity between BPA and the ER, which is much weaker (ca. 1/2000-fold) than that of the affinity between estradiol and the ER (Tsutsui et al., 2000). Following exposure to 600 μg/l BPA for 4 days, the expression of *ERα* is increased in liver, gonad, and brain tissue in *Rivulus marmoratus* (Seo et al., 2006), which is consistent with the results of our exposure experiment. Exposure to 8,000 μg/l BPA for 8 h can induce an increase in the expression of *ERα* in the liver tissue of male medaka; however, exposure to 800 μg/l BPA did not have this effect (Yamaguchi et al., 2005). In our experiment, exposure to 500 μg/l BPA significantly increased the expression of *TERα* in liver tissue, which suggests that *T. albonubes* is more sensitive to BPA than adult male medaka.

NP is a weak environmental estrogen. Exposure to different concentrations (5 μg/l, 15 μg/l, and 50 μg/l) of NP for 7 days increases the expression of *ERβ* in the liver and brain tissue of juvenile *S. salar* (Meucci and Arukwe 2006). The expression of *ERα* is increased in the liver tissue of medaka following exposure to 500 μg/l NP (Yamaguchi et al., 2005). The expression of ER genes is up-regulated in the liver tissue of juvenile *S. salar* following 4-NP exposure, and the expression of ER genes increases with the level of 4-NP exposure (Yadetie et al., 1999). 4-NP has been shown to inhibit the binding of E_2_ to the ER, which suggests that NP might induce the expression of ER genes via the ER pathway. The above findings are consistent with the results of our study. However, the expression of *ERα* and *Erβ* is down-regulated in various tissues in *R. marmoratus* following exposure to 300 μg/l NP for 4 days (Seo et al., 2006). The expression of *ERα*, *ERβ1*, and *ERβ*2 is reduced in *Gobiocypris rarus* following exposure to 10, 100, and 1,000 nM NP for 3 days (Wang et al., 2011). These varying sensitivities to NP might be explained by variation among species, reproductive periods, and seasons.

Changes in the expression of the proteins encoded by *TER* genes in testis tissue with the level of EDC exposure varied and were revealed by Western blotting. Variation in the expression of TER proteins among exposure groups was consistent with *TER* gene expression patterns; the only exception was for the expression of TER proteins in testis tissue in the EE_2_ exposure group. This suggests that the expression of ER genes and proteins can be induced by E_2_, BPA, and NP. The expression of three ER genes in the testis tissue of *T. albonubes* first increased and then decreased under EE_2_ exposure, and the expression of proteins increased with the level of EE_2_ exposure. This finding indicates that the stimulation of gene and protein expression during the response to EE_2_ exposure is not synchronized. However, exogenous substances affect the expression of genes of organisms, and the induction of the expression of these genes can lead to changes at the transcriptional and translational levels; generally, patterns of translational expression should follow patterns of transcriptional expression. The causes for the lack of synchronization in the expression of TER proteins and *TER* genes following EE_2_ exposure require further investigation. The expression of heat shock proteins in *T. albonubes* is not always consistent with the expression of the genes that encode these proteins (Jing et al., 2013). Thus, the effects of many EDCs on the expression of ER can vary, and their mechanisms of action are affected by various non-mutually exclusive factors. Changes in some conditions can potentially turn inhibitory effects into inductive effects and vice versa.

## Data Availability

The original contributions presented in the study are included in the article/supplementary material, further inquiries can be directed to the corresponding authors.
